# PAK1 Protein Expression in the Auditory Cortex of Schizophrenia Subjects

**DOI:** 10.1371/journal.pone.0059458

**Published:** 2013-04-22

**Authors:** Anthony J. Deo, Isaac M. Goldszer, Siyu Li, James V. DiBitetto, Ruth Henteleff, Allan Sampson, David A. Lewis, Peter Penzes, Robert A. Sweet

**Affiliations:** 1 Translational Neuroscience Program, Department of Psychiatry, University of Pittsburgh School of Medicine, Pittsburgh, Pennsylvania, United States of America; 2 Physician Scientist Training Program, University of Pittsburgh School of Medicine, Pittsburgh, Pennsylvania, United States of America; 3 Department of Statistics, University of Pittsburgh, Pittsburgh, Pennsylvania, United States of America; 4 Department of Neuroscience, University of Pittsburgh, Pittsburgh, Pennsylvania, United States of America; 5 Department of Physiology, Northwestern University Feinberg School of Medicine, Chicago, Illinois, United States of America; 6 Department of Psychiatry and Behavioral Sciences, Northwestern University Feinberg School of Medicine, Chicago, Illinois, United States of America; 7 Department of Neurology, University of Pittsburgh School of Medicine, Pittsburgh, Pennsylvania, United States of America; 8 VISN 4 Mental Illness Research, Education and Clinical Center, VA Pittsburgh Healthcare System, Pittsburgh, Pennsylvania, United States of America; Hertie Institute for Clinical Brain Research and German Center for Neurodegenerative Diseases, Germany

## Abstract

Deficits in auditory processing are among the best documented endophenotypes in schizophrenia, possibly due to loss of excitatory synaptic connections. Dendritic spines, the principal post-synaptic target of excitatory projections, are reduced in schizophrenia. p21-activated kinase 1 (PAK1) regulates both the actin cytoskeleton and dendritic spine density, and is a downstream effector of both kalirin and CDC42, both of which have altered expression in schizophrenia. This study sought to determine if there is decreased auditory cortex PAK1 protein expression in schizophrenia through the use of quantitative western blots of 25 schizophrenia subjects and matched controls. There was no significant change in PAK1 level detected in the schizophrenia subjects in our cohort. PAK1 protein levels within subject pairs correlated positively with prior measures of total kalirin protein in the same pairs. PAK1 level also correlated with levels of a marker of dendritic spines, spinophilin. These latter two findings suggest that the lack of change in PAK1 level in schizophrenia is not due to limited sensitivity of our assay to detect meaningful differences in PAK1 protein expression. Future studies are needed to evaluate whether alterations in PAK1 phosphorylation states, or alterations in protein expression of other members of the PAK family, are present in schizophrenia.

## Introduction

Recent studies have identified several electrophysiological, morphological and molecular changes in the auditory cortex of schizophrenia subjects. Individuals with schizophrenia have reduced auditory cortex gray matter volume [1]; [2] and deficits in auditory sensory processing. These deficits, evidenced by a reduced ability to discriminate pure tones, correlate with core negative symptoms of this illness such as impairments in detecting spoken emotional tone, in phonologic processing and in reading attainment [3]; [4]. Impaired tone discrimination is also correlated with reduced magnitude of Mismatch Negativity (MMN), an event-related potential arising after auditory stimuli that deviate from a repetitive stimulus in one characteristic (e.g. pitch) [5]; [6].

Tone discrimination depends on the primary auditory cortex (AI), contained within Heschel's gyrus (HG), which sharpens the frequency representations present at lower levels of auditory processing [7]; [8]. Similarly tuned and reciprocally connected [9]; [10] layer 3 pyramidal cells in AI excite each other, selectively amplifying the thalamocortical signal [8]. MMN similarly reflects activity within layer 3 circuits of AI, arising after the initial thalamic volley, and is dependent on excitatory neurotransmission [11]. A previous study identified a 27% reduction in density of a marker of dendritic spines, post-synaptic components of excitatory glutamatergic signaling, within deep layer 3 of AI in subjects with schizophrenia [12]. Spine density was correlated with the density of non-selectively labeled pre-synaptic axon boutons [12]; [13]. These findings likely contribute to the reduced auditory cortex gray matter volume in subjects with schizophrenia and to an impaired spread of activation within the layer 3 pyramidal cell networks of AI. A reduction in dendritic spine density has also been observed in the dorsolateral prefrontal cortex in post-mortem studies of schizophrenia [14]; [15].

Dendritic spines remain plastic structures, with a proportion arising and retracting into adulthood [16]–[18]. A net elimination of spines will result from reduced spine emergence, persistence and/or increased spine retraction. Formation and maintenance of dendritic spines is dependent on the organization and stabilization of an actin cytoskeleton [19]. Hence, disturbances in molecular pathways known to regulate actin stabilization may contribute to abnormalities in dendritic spine number and morphology.

Of those proteins known to regulate actin stabilization, p21-activated kinase 1 (PAK1) is a plausible point of dysregulation contributing to a reduction in dendritic spines in schizophrenia, a premise supported by multiple lines of evidence. First, PAK1, a serine/threonine protein kinase, alters F-actin stabilization. PAK1 activates LIMK which phosphorylates cofilin, inactivating it so that it cannot depolymerize f-actin [20]. Second, overexpression of either dominant negative or kinase dead PAK1 constructs in hippocampal neuron cultures reduced spine density [21]; [22]. Dominant negative PAK1 also altered spine morphology [21]. Overexpression of wild type PAK1 or constitutively active PAK1 resulted in increased spine density [21]; [22]. *In vivo* models of reduced PAK1 expression also provide evidence of altered spine function and morphology, although less consistent evidence of reduced spine density are present. PAK1 knockout mice have been shown to have deficient long term-potentiation in hippocampal region CA1, with reduced f-actin in spines, although reductions in spine density were not present [23]. In contrast, PAK1/PAK3 double knockouts show a decrease in synaptic density and bidirectional synaptic plasticity [24]. PAK1/PAK3 double knockout was also associated with reduced numbers of spines with mature morphologies, although there was not a change in overall spine density due to increases in spines with immature morphologies [24]. Third, PAK1 is dually regulated by both kalirin (via Rac1) and CDC42. Kalirin has been shown to influence dendritic spine number and morphology [25]; [26], while CDC42 has been demonstrated to be involved with filopodia formation and actin stabilization [27]. mRNA for the kalirin-7 isoform and for CDC42 have been reported to be reduced in schizophrenia [28]. More recently we examined protein levels of kalirin isoforms in the auditory cortex of subjects with schizophrenia, finding increased levels of the kalirin-9 isoform, while the more abundant kalirin-5, kalirin-7, and kalirin-12 isoforms were unchanged [29].

We hypothesized that altered PAK1 protein expression may contribute to the reduction in dendritic spine density in the auditory cortex of schizophrenia subjects. We utilized quantitative western blotting to measure PAK1 levels in post mortem auditory cortex gray matter samples from 25 schizophrenia subjects and matched controls in whom we had previously measured kalirin isoform levels [28].

## Results

### PAK1 protein expression is not altered in schizophrenia

**Figure 1:**
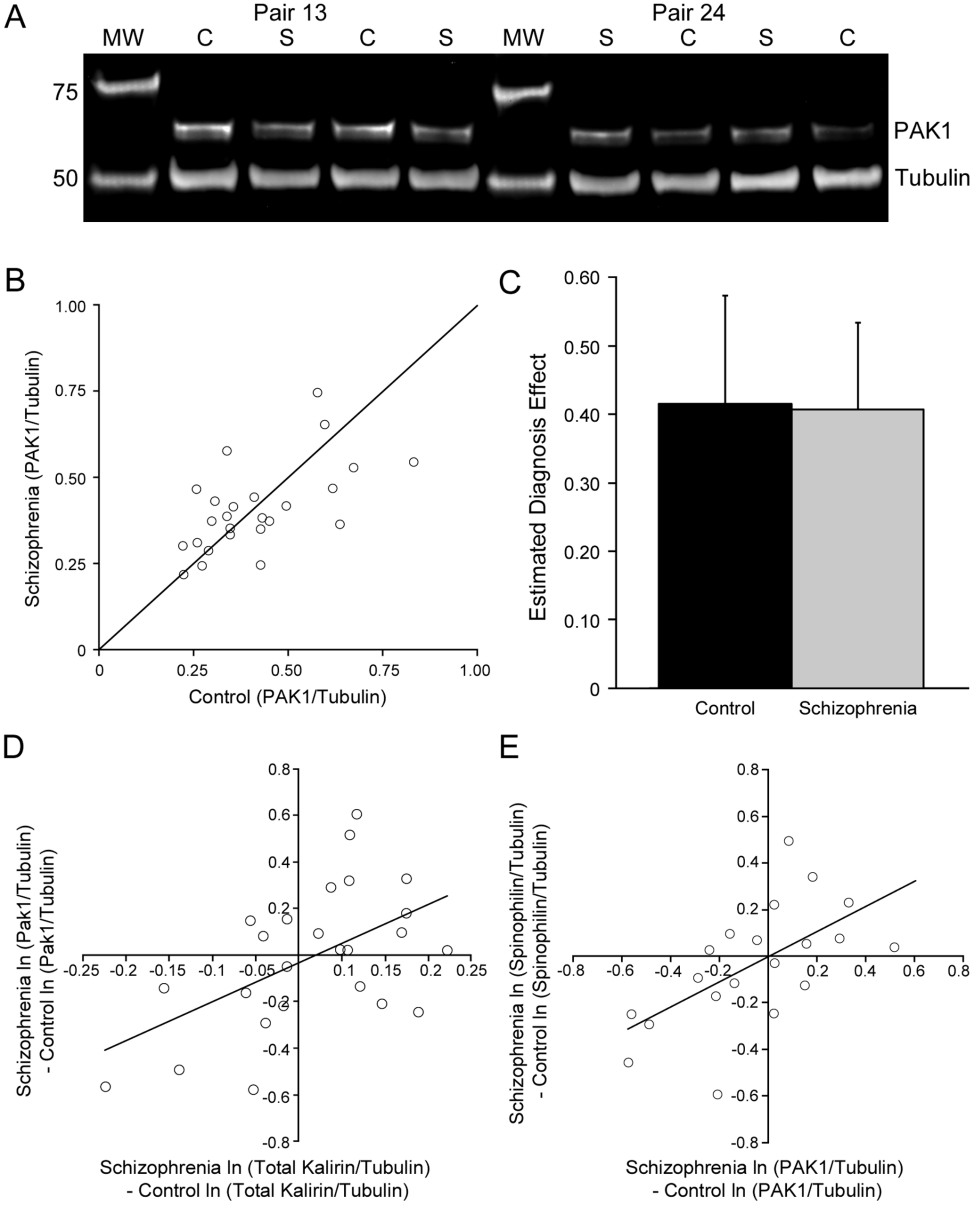
PAK1 Expression in schizophrenia. **A**) Detection of PAK1 in human gray matter extracts. Bands corresponding to the predicted molecular weights of PAK1 and Tubulin are readily detected in human gray matter extracts from subjects with schizophrenia (S) and matched control subjects (C). MW, Molecular Weight Markers. **B**) Comparison of PAK1 expression (normalized to tubulin expression) for the 25 pairs of subjects. Each point represents a pair of subjects. The diagonal reference line represents a control: schizophrenia ratio of one. Points above the line indicate increased expression in schizophrenia subjects, points below the line indicate decreased expression in schizophrenia subjects. **C**) Mean (SD) expression PAK1:tubulin in schizophrenia and control subjects. **D**) Correlation of within pair differences in log PAK1 expression with the corresponding measure of log total kalirin protein expression (r = 0.55, p = 0.004). **E**) Correlation of within pair differences in PAK1 expression with the corresponding measure of spinophilin protein expression (r = 0.61, p = 0.004).

PAK1 protein level was not significantly different between the schizophrenia subjects and matched controls in our cohort (t_22.7_ = −0.79, p = 0.44). The distribution of PAK1 level was evenly distributed among schizophrenia and control subjects with 48% of schizophrenia subjects and 52% of control subjects having higher PAK1 levels (Figure 1 A–C).

There was no significant interaction between PMI group (High, Medium and Low) and diagnosis (F_2, 28.1_ = 1.30, p = 0.29). The 95% nominal confidence intervals for the diagnostic difference in the High, Medium and Low PMI groups were (−0.19, 0.38), (−0.18, 0.28) and (−0.51, 0.11), indicating no effect of diagnosis in any of these subgroups.

**Figure 2:**
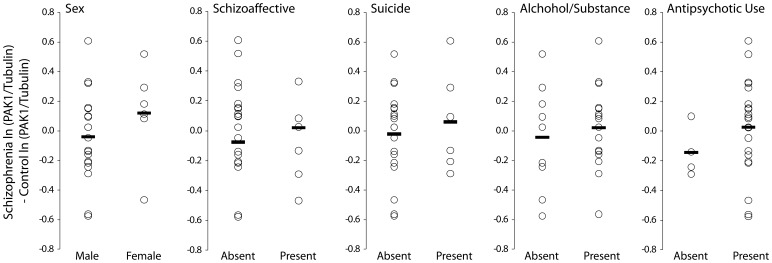
Effect of subject variables on PAK1 expression in schizophrenia. Each point represents the mean within pair difference in log PAK1 expression. Horizontal bars are group means. No differences were significant.

We further examined whether PAK1 level (log scale) was selectively altered in additional subgroups of subjects (Figure 2). There was no difference in PAK1 levels between subject pairs when separated by sex (t_23_ = −1.15, p = 0.26), diagnosis of schizoaffective disorder (t_23_ = −0.66, p  = 0.52), death by suicide (t_22.9_ = 0.56, p = 0.58), history of substance use disorder (t_23_ = 0.49, p = 0.63), or antipsychotic use at time of death (t_22.8_ = 1.02, p = 0.32). There was no significant correlation of the pairwise change in PAK1 levels with age of onset (t_23.1_ = −0.17, p = 0.87) or duration of schizophrenia (t_23_ = 0.33, p = 0.74).

We had previously determined relative protein levels of kalirin isoforms within auditory cortex gray matter from these subject pairs; the within subject-pair differences in log of PAK1 protein levels and log of total kalirin protein levels were positively correlated (r = 0.55, p = 0.004, Figure 1), although neither PAK1 nor total kalirin protein level (unpublished observation) significantly differed between schizophrenia and control subjects.

We similarly evaluated whether PAK1 protein levels were correlated with protein levels of spinophilin, a marker of dendritic spines,[30]; [31] sensitive to changes in spine density.[12]; [32] Spinophilin protein levels were obtained in the same individuals. However, because of evidence for loss of spinophilin immunoreactivity when postmortem intervals were greater than 24 hours in an animal model (Figure S1) we restricted these analyses to the 20 pairs of subjects with observed postmortem intervals≤24 hours (Table 1). Spinophiln protein levels, like PAK1 levels, were unchanged in subjects with schizophrenia (t_18_ = 0.58, p = 0.57). Nevertheless, PAK1 protein levels and spinophilin protein levels were positively correlated among all subjects (Figure S2, r = 0.37, p = 0.02). A stronger correlation was seen for the within subject-pair differences in log PAK1 protein levels and differences in log spinophilin protein levels (Figure 1, r = 0.61, p = 0.004).

**Table 1:** Characteristics of control subjects and subjects with schizophrenia.

	Control									Schizophrenia								
Pair	Case	S/R/A	PMI (hrs) [Obs]	Storage Time (mos)	COD	Diagnosis	pH	Hand	Meds ATOD	Case	S/R/A	PMI (hrs) [Obs]	Storage Time (mos)	COD	Diagnosis	pH	Hand	Meds ATOD
1*	1326	M/W/58	16.4 [16.4]	62	ASCVD	None	6.7	R	O	1453	M/W/62	11.1 [11.1]	39	Trauma	Paranoid schizophrenia; ADR	6.4	R	BO
2*	1247	F/W/58	22.7 [22.7]	77	ASCVD	None	6.4	R	O	1240	F/B/50	22.9 [20.2]	78	ASCVD	Undifferentiated schizophrenia; ADR	6.3	R	OP
3	1086	M/W/51	24.2 [23.8]	97	ASCVD	None	6.8	R	N	10025	M/B/52	27.1 [27.1]	76	ASCVD	Disorganized schizophrenia; OAR	6.7	R	N
4	10005	M/W/42	23.5 [23.5]	84	Trauma	None	6.7	R	O	1256	M/W/34	27.4 [24.8]	76	Hanging	Undifferentiated schizophrenia	6.4	R	P
5	1255	M/B/37	22.0 [21.8]	76	Pulmonary embolism	None	5.9	R	O	10020	M/W/38	28.8 [28.8]	77	Salicylate overdose	Paranoid schizophrenia; AAC; OAC	6.6	R	CDP
6*	1480	M/W/62	20.1 [19.8]	36	ASCVD	Reading D/O	6.7	R	O	1263	M/W/62	22.7 [22.7]	75	Asphyxiation	Undifferentiated schizophrenia; ADR	7.1	R	DP
7*	1119	M/W/57	20.4 [20.4]	91	ASCVD	None	6.8	R	N	1173	M/W/62	22.9 [18.8]	86	ASCVD	Disorganized schizophrenia; ADR	6.4	R	OP
8*	1317	M/W/56	22.9 [22.9]	64	ASCVD	None	6.5	L	O	1361	M/W/63	23.2 [23.2]	57	Cardio-myopathy	Schizoaffective disorder; ODC	6.4	U	COP
9*	1307	M/B/32	4.8 [4.8]	67	ASCVD	None	6.7	R	N	10024	M/B/37	6.0 [6.0]	76	ASCVD	Paranoid schizophrenia	6.1	L	O
10*	1067	M/W/49	6.5 [6.5]	99	ASCVD	None	6.6	R	O	1296	M/W/48	7.8 [7.8]	68	Pneumonia	Undifferentiated schizophrenia	6.5	L	DOP
11*	1196	F/W/36	14.5 [14.5]	85	Asphyxiation	None	6.4	R	O	1211	F/W/41	20.1 [20.1]	83	Sudden unexpected death	Schizoaffective disorder	6.3	L	DOP
12*	1099	F/W/24	9.1 [9.1]	96	Cardio-myopathy	None	6.5	R	O	10023	F/B/25	20.1 [20.1]	77	Drowning	Disorganized schizophrenia	6.7	R	BDP
13*	806	M/W/57	24.0 [24.0]	145	Pulmonary embolism	None	6.9	R	O	665	M/B/59	28.1 [12.9]	170	Intestinal hemorrhage	Paranoid schizophrenia; ADC	6.9	R	DOP
14*	739	M/W/40	15.8 [15.8]	162	ASCVD	None	6.9	R	N	1088	M/W/49	21.5 [21.5]	99	Combined drug overdose	Undifferentiated schizophrenia; ADC; OAC	6.5	R	DOP
15	822	M/B/28	25.3 [25.3]	146	ASCVD	None	7.0	L	N	787	M/B/27	19.2 [19.2]	152	Gunshot	Schizoaffective disorder; ODC	6.7	L	OP
16*	727	M/B/19	7.0 [7.0]	160	Trauma	None	7.2	R	N	829	M/W/25	5.0 [5.0]	141	Salicylate overdose	Schizoaffective disorder; ADC; OAR	6.8	U	BC
17*	659	M/O/46	22.3 [22.3]	170	Peritonitis	None	6.9	R	N	930	M/W/47	15.7 [15.7]	120	ASCVD	Disorganized schizophrenia; ADR; OAR	6.2	R	COP
18*	852	M/W/54	8.0 [4.8]	136	Cardiac Tamponade	None	6.8	R	N	722	M/B/45	9.1 [8.3]	161	Upper GI bleed	Undifferentiated schizophrenia; ODR;OAR	6.7	R	OP
19*	685	M/W/56	14.5 [13.4]	169	Hypoplastic coronary artery	None	6.6	R	O	1105	M/W/53	7.9 [7.9]	95	ASCVD	Schizoaffective disorder	6.2	R	P
20*	686	F/W/52	22.6 [20.4]	167	ASCVD	None	7.0	R	O	802	F/W/63	29.0 [19.9]	146	Right ventricular dysplasia	Schizoaffective disorder; ADC; ODR	6.4	M	COP
21*	1092	F/B/40	16.6 [16.6]	95	Mitral valve prolapse	None	6.8	R	O	1010	F/B/44	18.7 [18.7]	108	Sudden unexpected death	Undifferentiated schizophrenia	6.2	L	CDP
22*	1488	M/B/39	21.5 [21.5]	33	Pulmonary embolism	None	6.4	R	N	1222	M/W/32	30.8 [18.6]	79	Combined drug overdose	Undifferentiated schizophrenia; AAC	6.4	R	DP
23*	1047	M/W/43	13.8 [13.8]	101	ASCVD	None	6.6	R	O	933	M/W/44	8.3 [8.3]	119	Myocarditis	Disorganized schizophrenia	5.9	U	CDOP
24	700	M/W/42	26.1 [26.1]	164	ASCVD	None	7.0	R	N	625	M/B/49	23.5 [23.5]	174	ASCVD	Disorganized schizophrenia; AAC	7.3	R	DOP
25*	818	F/W/67	23.5 [23.5]	144	Anaphylactic reaction	None	7.1	R	O	917	F/W/71	23.8 [23.1]	123	ASCVD	Undifferentiated schizophrenia	6.8	U	OP
	N (%) or Mean (SD)	M: 19 (76) Age: 45.8 (12.3)	17.9 (6.6)	109 (43)			6.7 (0.3)	R: 23 (92)			M: 19 (76) Age: 47.3 (12.9)	19.2 (8.1)	102 (37)			6.5 (0.3)	R: 15 (60)	

665: Min (12.9) Max (43.2) Avg (28.1) **One Pair Not Shown: HU 1201 (C) and 1189 (S) 802: Min (19.9) Max (37.9) Avg (29) 1222: Min (18.6) Max (43) Avg (30.8)

All subject pairs shown were included in analyses of PAK1 and kalirin protein levels. Subject pairs also analyzed for spinophilin protein levels (Observed PMI≤24 hours) are indicated by an asterisk. Control and schizophrenia subjects significantly differed in pH and handedness, but not in age, sex, PMI, and tissue storage time. S/R/A, Sex/Race/Age; PMI, postmortem interval (calculated as lag from midpoint of time last seen alive – time discovered dead to time of brain fixation. [Observed] is calculated as time discovered dead to time of brain fixation); COD, cause of death; Hand, handedness; Meds ATOD, medications used at time of death; ADC, Alcohol Dependence, current at time of death; ADR, Alcohol Dependence, in remission at time of death; AAC, Alcohol Abuse, current at time of death; AAR, Alcohol Abuse, in remission at time of death; ODC, Other Substance Dependence, current at time of death; ODR, Other Substance Dependence, in remission at time of death; OAC, Other Substance Abuse, current at time of death; OAR, Other Substance Abuse, in remission at time of death; L, Left; M, Mixed; R, Right; U, Unknown; B, Benzodiazepines; C, Anticonvulsants; D, Antidepressants; L, Lithium; N, No medications; O, Other medication(s); P,Antipsychotic; U, Unknown.

### Post mortem interval does not affect PAK1 protein expression

**Figure 3:**
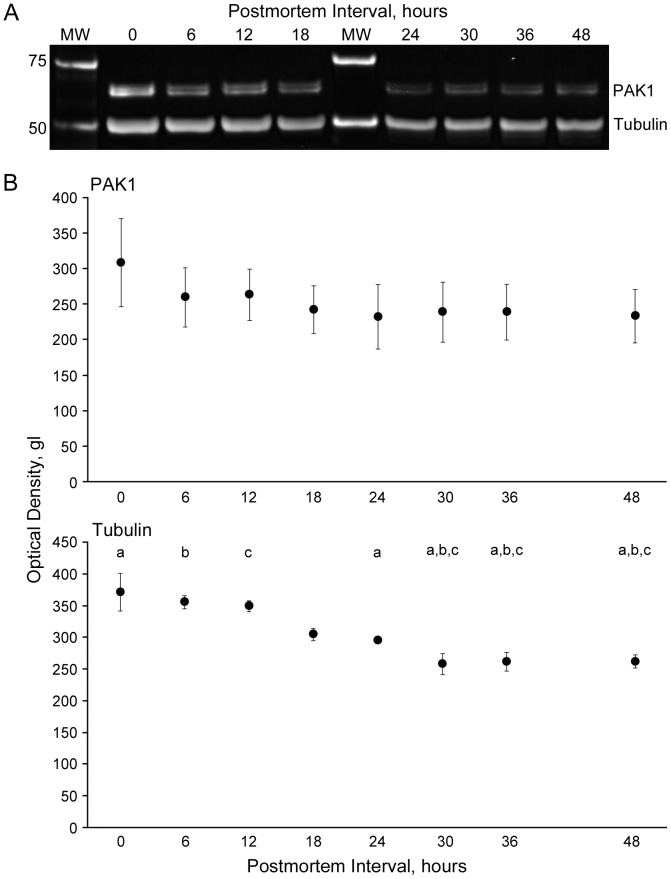
Effects of postmortem interval on PAK1 expression. **A**) Example western blot of PAK1 and Tubulin in mice in which the interval from sacrifice to brain harvesting was experimentally varied between 0 and 48 hours. MW, Molecular Weight Markers. **B**) Optical densities for PAK1 and Tubulin in mice in which the interval from sacrifice to brain harvesting was experimentally varied between 0 and 48 hours. Two sets of mice (each set containing one mouse for each PMI) were tested. Sets were run concurrently on separate gels, and the run was repeated. Mean (SEM) values for the four observations at each time point are shown. Time points sharing the same superscript letter differ significantly.

Failure to detect differences between groups in postmortem studies can occur because the measured protein, although detectable, is present at such a reduced level in comparison to the *in vivo* state that identifying further reduction is not possible, i.e. a “floor effect”. We thus examined the effect of post mortem interval on PAK1 level in cortical grey matter in a mouse model (Figure 3). We found that the effects of post mortem interval on PAK1 level (F_7,24_ = 0.35, p = 0.92) were minimal in the PMI range utilized in this study (PMI≤30 hours). There was a small, but statistically significant effect of PMI on β tubulin level (F_7,24_ = 9.56, p≤0.01), however, this is readily accounted for by subject matching which includes PMI. In the human study there were no significant effects of PMI on PAK1 level (F_1,31.9_ = 0.76, p = 0.39), although we did detect the previously mentioned nominally significant interaction between PMI and diagnosis.

## Discussion

The emergence and persistence of dendritic spines is likely regulated by numerous molecular pathways with complex interactions. Strong potential candidates contributing to dendritic spine abnormalities in schizophrenia will demonstrate regulatory effects on the actin cytoskeleton, dendritic spine number and spine morphology, as well as interactions with regulatory proteins whose expression are known to be altered in the disease state. While PAK1 fulfills all of these criteria and is stable in postmortem tissue, this study demonstrated that there is no change in PAK1 protein expression in whole grey matter extracted from the auditory cortex of schizophrenia subjects in our cohort.

This study has several strengths of that enhance confidence in the results. We evaluated a moderately large human tissue cohort, with sudden causes of death and indices of excellent tissue preservation. Initial studies established the linearity of our assay conditions allowing interpretation of the relative change in fluorescence intensities. Subjects with schizophrenia and matched control subjects were processed in pairs through all stages of the assay, including tissue harvesting, protein extraction and measurement, and western blotting. Since gel effects in western blotting can be substantial, this latter design element in which pairs of subjects were present in adjacent lanes, with replicates of each pair both within and across gels, was essential to reduce variability in our diagnostic comparison. Moreover, we used analytic models that explicitly addressed these nested repeated comparisons, enhancing the precision of our estimate of the diagnosis effect. Using this approach in these subjects, we had previously found an increased protein level of kalirin-9, indicating that our approach is sufficiently sensitive to detect protein changes.

Although there was no overall change in mean PAK1 protein levels in our subjects with schizophrenia, within pair differences in PAK1 levels were correlated with those of an upstream effector, total kalirin, and with levels of a dendritic spine marker, spinophilin. Detection of these correlated changes further suggests that our approach was sufficient to detect potential biologic effects. Similarly, evaluation of postmortem effects on PAK1 levels in a mouse model showed minimal impact for the intervals represented within our cohort, indicating that the failure to find a change in PAK1 was not likely an artifact of “floor effects” in the human postmortem cohort. One potential limitation of our study is the lack of evaluation of the potential influences of antipsychotic use on PAK1 levels in a model system, however, our analysis did not show an effect of antipsychotic use at the time of death in our human cohort.

**Figure 4:**
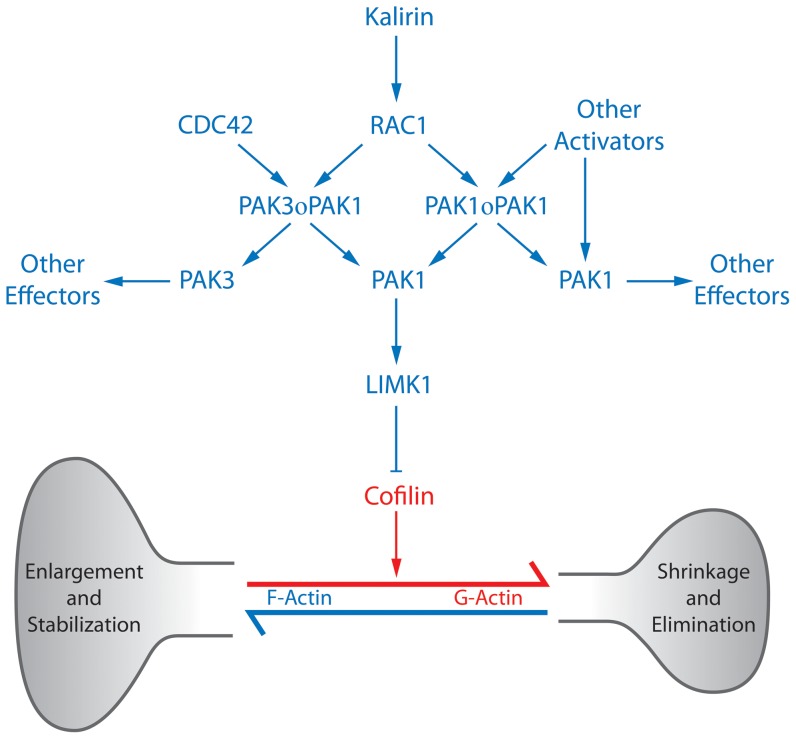
PAK1 Signaling Pathway. PAK1 resides in inactive homo- or heterodimers. Binding of the Rho-GTPase, RAC1 (or CDC42) causes dissociation of the dimers and activation of PAK1. PAK1 activates LIMK1, which inhibits cofilin-mediated f-actin depolymerization. PAK1 may be subject to activation by non-GTPase mechanisms, and can activate other effector pathways, some of which (e.g. MLCK and MLC) may also impact spine dynamics. Blue indicates promotion of dendritic spine persistence, red indicates promotion of spine elimination.

Our finding of no change in PAK1 protein expression in the auditory cortex in combination with a recent study that found no change in PAK1 levels in the anterior cingulate cortex, but increased PAK1 levels in the dorsolateral prefrontal cortex, in subjects with schizophrenia, suggests there may be regional differences in disease-related PAK1 expression.[33] Alternatively, the significant correlation of within pair reductions in PAK1 and spinophilin raises the possibility that it is a subset of subjects with schizophrenia that have deficits in signaling via PAK1 to dendritic spines. Figure 4 summarizes the major PAK1 signaling pathway regulating actin dynamics and dendritic spines. PAK1 activity is regulated by the Rho GTPases, Rac1 and CDC42, which in their active GTP bound state bind to PAK1 dimers inducing a conformational change which removes the autoinhibitory domain from the kinase active site [34]. These conformational changes promote autophosphorylation at threonine 423 in the active site, which is required to maintain the uninhibited state and to promote full catalytic activity. In addition at least two other phosphorylation sites exist which promote and maintain catalytic activity regulated by numerous additional mediators (ie. Cdk-5, PDK1), thus creating a number of possible differentially phosphorylated, catalytically active states [34]. Prior studies have demonstrated enrichment of the phospho-threonine 423 PAK1 in the post-synaptic density of mouse cortical neurons [35]; [36]. It is plausible that while PAK1 protein expression is not changed, the fraction of PAK1 in its active phosphorylated state is altered. Measurement of levels of these specific phosphorylation states, through the use of phospho-specific antibodies, may provide more biologically relevant indications of dysregulation of this molecular cascade. However, phosphorylation states vary with postmortem time and conditions, making determination of individual phosphorylation states in post-mortem samples challenging [37]. Nevertheless, one recent study identified reduced levels of phospho-threonine 423 PAK1 in the dorsolateral prefrontal cortex and anterior cingulate cortex of subjects with schizophrenia [33].

Additional consideration must be given to the use of protein extracted from whole gray matter in this study, which does not allow for differentiation of PAK1 levels in individual layers or specific cellular compartments. Prior findings of altered kalirin protein levels and kalirin and Cdc42 mRNA expression in schizophrenia were not layer specific [28]; [29], leading us to evaluate whole gray matter. Nevertheless, it remains possible a selective decrease in PAK1 protein level within specific layers may contribute to spine loss. Such a specific change may not be detectable in the overall background of PAK1 protein expression and would require separate studies to detect changes confined to individual layers. Similarly, PAK1 levels may be selectively altered in the spine compartment, and may require measurement specifically in this compartment. Finally, other members of the PAK family may be relevant to spine density. For instance, a rare chromosomal deletion including the *PAK2* gene has recently been associated with schizophrenia [38]. Also of note, mutations in the *PAK3* gene, discovered to be associated with nonsyndromic mental retardation, have been noted to alter spine number through a CDC42 mediated pathway [39]. Recent data indicate that PAK1 protein and PAK3 protein colocalize within dendritic spines where they heterodimerize, regulating PAK3 signaling. Of interest, this dimerization is inhibited by mutations in PAK3 that lead to mental retardation, suggesting that PAK1 levels may contribute to spine reductions through this mechanism [40]. Thus, while we found no change in overall PAK1 protein expression in schizophrenia, further exploration of possible changes in both biochemical properties and compartmental localization of members of the PAK family of proteins will be required to fully evaluate whether they play a role in dendritic pathology in schizophrenia.

## Materials and Methods

### Ethics Statement

All brain specimens were obtained during autopsy after obtaining a witnessed verbal consent from the next of kin, using a procedure reviewed and approved by the University of Pittsburgh Committee for Oversight of Research and Clinical Training Involving Decedents. Verbal consent was audiotaped and a written document summarizing the consent process was generated and signed by the individual obtaining consent and an independent witness. Family members of decedents provided written consent to participate in a postmortem research diagnostic interview as approved by the University of Pittsburgh Institutional Review Board. All data were analyzed anonymously. Animal studies were approved by the University of Pittsburgh Institutional Animal Care and Use Committee.

### Human Subjects

A total of 25 subjects with a diagnosis of either schizophrenia or schizoaffective disorder were each matched to a control subject for sex, as closely as possible for age and post-mortem interval (PMI), and to the extent possible, for handedness (Table 1). All brain specimens were obtained during autopsy at the Allegheny County Medical Examiner's Office after obtaining consent from the next of kin. An independent panel of experienced clinicians made consensus DSM-IV diagnoses using a previously described method [14] approved by the University of Pittsburgh Institutional Review Board. The right hemisphere was blocked coronally at 1–2 cm intervals and the resultant slabs snap frozen in 2-methyl butane on dry ice, and stored at −80°C.

### Sample Preparation

**Figure 5:**
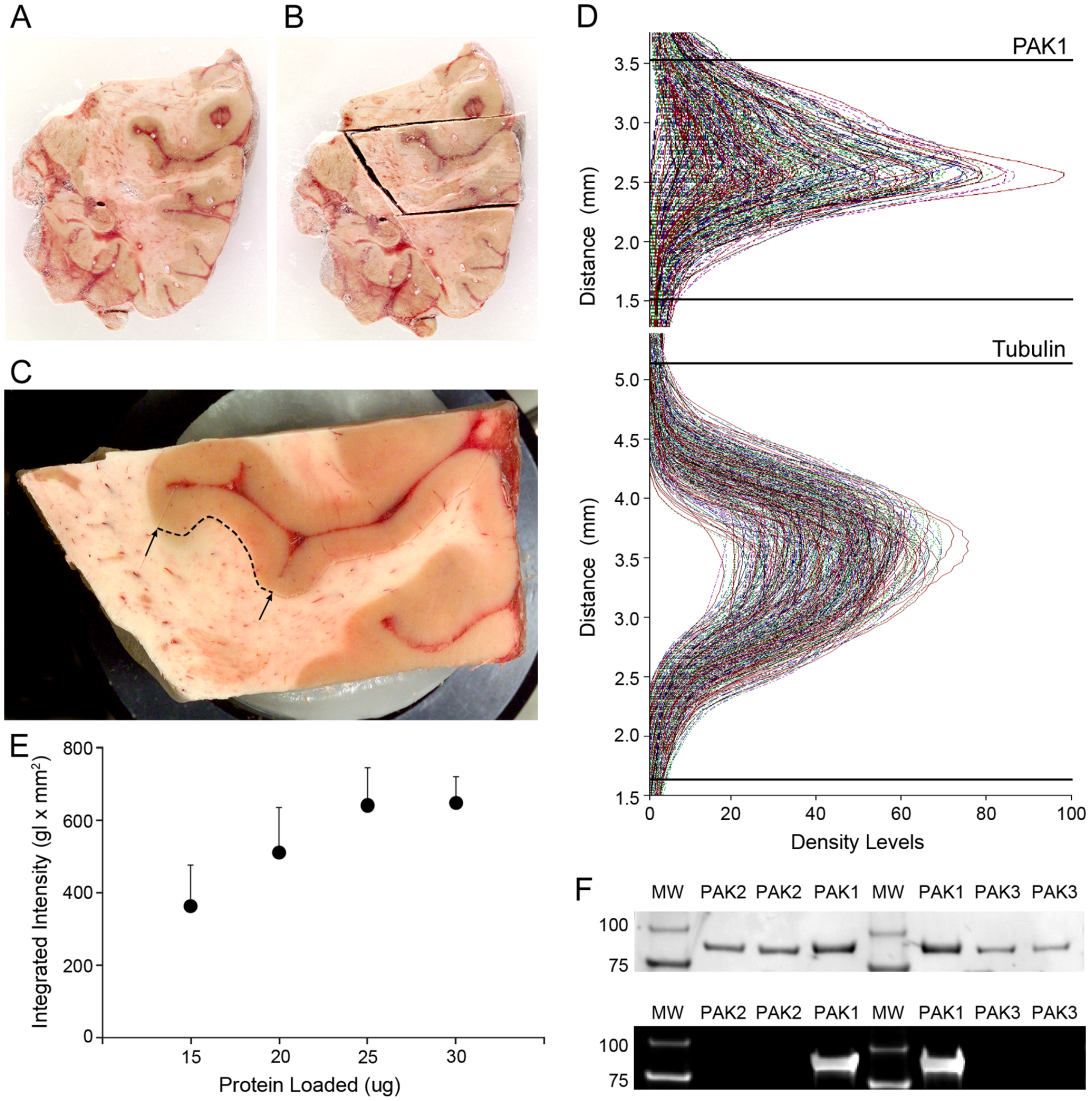
Processing of human tissue for PAK1. **A**–**C**) show an example of a frozen coronal slab through the right temporal lobe from which a block containing the STG and Heschl's gyrus (located between the arrows in **C**) has been excised and mounted for cryostat sectioning. The boundary between the gray and white matter of Heschl's gyrus (dotted line) was undercut so that only gray matter was collected for protein extraction. **D**) Demonstrates our approach to quantification of PAK1 and tubulin. Lanes from all blots were simultaneously imported into the analysis software, and for quantification of each protein the peaks were aligned, and then a single band definition applied to all blots concurrently, indicated by the black horizontal lines. **E**) PAK1 optical density as a function of micrograms of protein loaded per lane. The mean (SD) of 3 human subjects, assayed together in duplicate runs is shown. It can be seen that the protein loading used in the comparison of schizophrenia and control subjects (20 µg) sits within the linear detection range. **F**) Western blot demonstrating isoform specificity of anti-PAK1 antibody. Lanes were loaded with 0.5 µg of each full length GST-tagged recombinant protein (ProQinase # 0357-0000-1, 0304-0000-1, 0422-0000-1, Freiburg, Germany), and detected with Coomassie Blue (top) or with anti-PAK1 antibody (bottom) as described for human and mouse studies.

Tissue slabs containing the superior temporal gyrus (STG) with an evident Heschl's Gyrus (HG) located medial to the planum temporal were identified and matched on rostral-caudal level within pairs [41]; [42]. From these slabs, the STG was removed as a single block (Figure 5 A–C) and the area of HG gray matter was estimated using Image J [43]. After undercutting the gray matter, a total of 30 mm^3^ (approximating 30 µg) of grey matter was collected from HG by taking 40 µm sections, and frozen at −80C. Total protein was extracted using SDS extraction buffer (0.125 M Tris-HCl (pH 7), 2% SDS, and 10% glycerol) at 70 °C. Protein concentration was estimated using a bicotinic acid assay (BCA™ Protein Assay Pierce # 23225). Pairs were run together, and assayed in triplicate. The final protein concentration utilized for each sample was the mean of the triplicate runs.

### Western Blotting

This study consists of 25 matched pairs of control and schizophrenia subjects examined in 13 runs, with 2 pairs per run for 12 runs and 1 pair for 1 run with each run consisting of 4 gels. For the 12 runs with 2 pairs in each run, each gel contained two different pairs loaded using a total of 8 lanes so that the control member of each pair appears on 2 lanes and the schizophrenia member of each pair appears on 2 lanes, with members of a pair run in adjacent lanes. For the run with 1 pair, each gel was loaded using a total of 4 lanes so that the control and schizophrenia member of that pair appears on 2 adjacent lanes. The experimenter was blind to diagnosis during experimentation and quantification of blots. Pilot studies were used to establish conditions providing for linear detection of all target proteins (Figure 5 E). Based on these studies, 20 mg of protein was aliquoted in 1× LI-COR Protein Loading Buffer (Licor Inc. Lincoln, Nebraska, USA), loaded on 4–20% SDS-PAGE gradient gels (Thermo Scientific, Rockford, Illinois, USA), and separated for 2 hours at room temperature in 1× SDS running buffer (Pierce 20× Tris Hepes SDS Buffer) at 75 V. Samples were then transferred to 0.45 µm PVDF (Millipore, Billerica, Massachusetts, USA)] in 1× Tris Glycine Blotting Buffer (Pierce) at 85 V for 50 minutes at 4°C. Membranes were incubated for 1 hour in Odyssey LiCor Blocking Buffer diluted 1∶1 in 1× TBS. The membrane was incubated overnight in PAK1 primary antibody (rabbit anti-PAK1, Invitrogen, 71–9300, Camarillo, Ca 93012) diluted 1∶1000 and mouse anti-β tubulin (Millipore# 05–661) diluted 1∶60,000, in Pierce SuperBlock blocking buffer with 0.1% Tween 20 (Sigma-Aldrich, St. Louis, Missouri, USA). The anti-PAK1 antibody did not detect PAK2 or PAK3 (Figure 5 F). Membranes were then incubated in LiCor IRDye secondary antibodies (goat anti-rabbit 800 nm; goat anti-mouse 680 nm) 1∶10,000 in Odyssey Licor Blocking Buffer diluted 1∶1 with TBS (0.1% Tween 20+0.02% SDS). Blots were scanned dry and bands detected using a Li-Cor Odyssey Infrared Scanner set at a resolution of 42 mm and the highest image quality. Procedures for spinophilin (detected using rabbit anti-spinophilin, Millipore # AB5669, Millipore, Billerica, Massachusetts, USA) diluted 1∶2000 were identical, with the exception that 10 µg of protein was loaded based on pilot studies to determine conditions providing for linear detection of spinophilin (Figure S1).

### Quantification of Western Blots

Images were quantified using MCID Core Version 7.0 (InterFocus Imaging Ltd., Linton, Cambridge, UK). The peaks for PAK1, spinophilin, and β tubulin on the histogram were independently aligned to a single point for all lanes from all blots by translating each lane along the distance axis. Once aligned, a band definition encompassing the full range of each band was applied uniformly to all lanes from all blots in the study on the histogram for each protein (Figure 5 D). The integrated intensity (mean intensity × mm^2^) and maximum intensity was acquired for each protein.

### Statistical Analyses

The response variable for the analysis was the natural logarithm of the ratio of PAK1 protein integrated intensity to the integrated intensity of tubulin, a normalizing protein that does not differ between diagnostic groups (t_22.5_ = 1.16, p = 0.26). For each subject, up to 8 measurements of this log ratio were made after excluding any measurements with gel artifacts. Each analysis used two linear mixed models, in which subject and gel were treated as random effects due to the fact that the log ratio measurements are repeated within each subject and within each gel. The primary model treated diagnosis, pair nested in run, and run as fixed effects, while tissue storage time and brain PH were treated as covariates. The secondary model ignored subject pairings and instead replaced the fixed effect of pair nested in run by the covariates gender, age, and PMI with tissue storage time and brain PH as additional covariates. (For the primary model, run*diagnosis interaction was not significant (F_12, 10.1_ = 0.31, p = 0.97), thus not included in the model. For the secondary model, there were no significant interactions between diagnosis and age (F_1, 30.4_ = 0.06, P = 0.80) or diagnosis and sex (F_1, 30.9_ = 0.86, P = 0.36), thus neither of them was included in the model). We did however identify a marginally significant interaction between diagnosis and PMI (F_1, 30_ = 3.31, P = 0.079). To further evaluate this we categorized the PMIs into Low (<14), Medium (14–23) and High (>23) and then examined the PMI*diagnosis interaction in the secondary model, now treating PMI as a categorical variable, and estimated the diagnostic effects in the three PMI groups.

To guard against bias in our band definitions, we also evaluated an alternate response variable, the natural logarithm of the ratio of the maximum intensities of PAK1 and tubulin. Similar primary and secondary analyses were done for this alternate variable and provided results consistent with the analysis utilizing the integrated intensity level so are not reported further.

The relationship of the natural logarithm of the tubulin normalized PAK1 ratios within each pair to each of the following confounds was also examined: schizoaffective diagnosis (yes/no), suicide as cause of death (yes/no), antipsychotic use at time of death (yes/no), history of substance use disorder (yes/no), age of onset, and duration of schizophrenia. Log tubulin normalized ratios for PAK1 on each gel were obtained by averaging PAK1 and Tubulin levels of Schizophrenia subject and Control subject over the gel separately, getting two Tubulin normalized PAK1 values, [PAK1_SCHZ_/Tubulin_SCHZ_] and [PAK1_CNTL_/Tubulin_CNTL_], and then getting the log ratio of the two Tubulin normalized PAK1 values. In computing the log tubulin normalized ratios for a subject, if a PAK1 (or tubulin) measurement was missing on one lane, the corresponding tubulin (or PAK1) measurement was also treated as missing on that lane. The analyses used linear mixed models to model the log tubulin normalized PAK1 ratio, where each of the confounds was treated as a fixed effect, and where gel and pair were treated as random effects in order to account for the four gels per pair and the two pairs per gel.

We examined the correlation of the pairwise differences (the differences of averaged log tubulin normalized ratios between Schizophrenia subject and Control subject within a pair where the average is over the four gels) for PAK1 with similarly calculated pairwise difference for total kalirin protein levels (as all 4 kalirin isoforms present in human cortex have Rac1 guanine nucleotide exchange factor activity and thus are likely to be upstream of PAK1 [44]). The log tubulin normalized ratios were computed for spinophilin using the same method. Furthermore, we evaluated the correlation of the pairwise difference in PAK1 values with the pairwise difference in spinophilin values, and also the correlation between corresponding PAK1 and spinophilin values among all subjects.

All tests were two-sided and conducted at the 0.05 significance level. The p-values for diagnostic group effect are based on the contrast of control effect minus schizophrenia effect. All analyses were implemented in SAS PROC MIXED (Version 9.2, SAS Institute Inc., Cary, NC).

### Animals

For examination of effects of PMI on PAK1, spinophilin, and β tubulin, 16 week old male C57Bl/6J mice (Jackson Laboratory, Bar Harbor, ME) were euthanized by CO_2_ inhalation and cervical dislocation. PMI in our human cases consists of two temperature components. From death until the time of discovery and transport to the medical examiner, cases are typically at room temperature. After arrival at the morgue they are stored under refrigeration until the time of autopsy (commonly the next morning). To model these components in mice we chose a fixed room temperature incubation followed by a variable period of refrigeration, as this most closely resembles the situation for our human tissue. After sacrifice, for 0 hr PMI mice brains were immediately extracted. All other animals were stored until brain extraction at room temperature for 4 hrs, then at 4°C. For all PMI time points, at the time of extraction brains were first bisected mid-sagittally with the left half fixed for 48 hrs in 4% paraformaldehyde and the right half cut in 1 mm coronal slabs and frozen at −80C. Total protein was extracted and assayed, and PAK1, spinophilin, and β tubulin quantified by western blot as described above.

## Supporting Information

Figure S1 **Spinophilin Western Blot Characterization and Validation. A**) Spinophilin immunoreactivity in human, wild type mouse (+/+) and spinophilin knockout mouse (−/−) demonstrate specificity of spinophilin detection. **B**) Spinophilin optical density as a function of micrograms of protein loaded per lane. The mean (SD) of a eight repeated assays of a human subject is shown. It can be seen that the protein loading used in the comparison of schizophrenia and control subjects (10 µg) sits within the linear detection range. **C**) Optical densities for spinophilin in mice (N = 2) in which the interval from sacrifice to brain harvesting was experimentally varied between 0 and 48 hours. Mean (SEM) value at each time point is shown. Time points sharing the same superscript letter differ significantly. **D**) Detection of spinophilin in auditory cortex gray matter extracts from two subject pairs. S, subjects with schizophrenia, C, matched control subjects.
(TIF)

Figure S2 **Correlation of PAK1 and Spinophilin Protein Levels within Subjects.** Filled circles represent subjects with schizophrenia, open circles represent normal control subjects. Black line is the regression line for schizophrenia subjects (r = 0.43, p = 0.057). Dashed line is the regression line for control subjects (r = 0.30, p = 0.19). Gray line is the regression line for both groups combined (r = 0.37, p = 0.02).
(TIF)
