# Supplementary figures and images for: PAK1 Protein Expression in the Auditory Cortex of Schizophrenia Subjects
Source: PLoS One. 2013 Apr 22;8(4):e59458. doi: 10.1371/journal.pone.0059458 (PMC3632562; doi:10.1371/journal.pone.0059458)

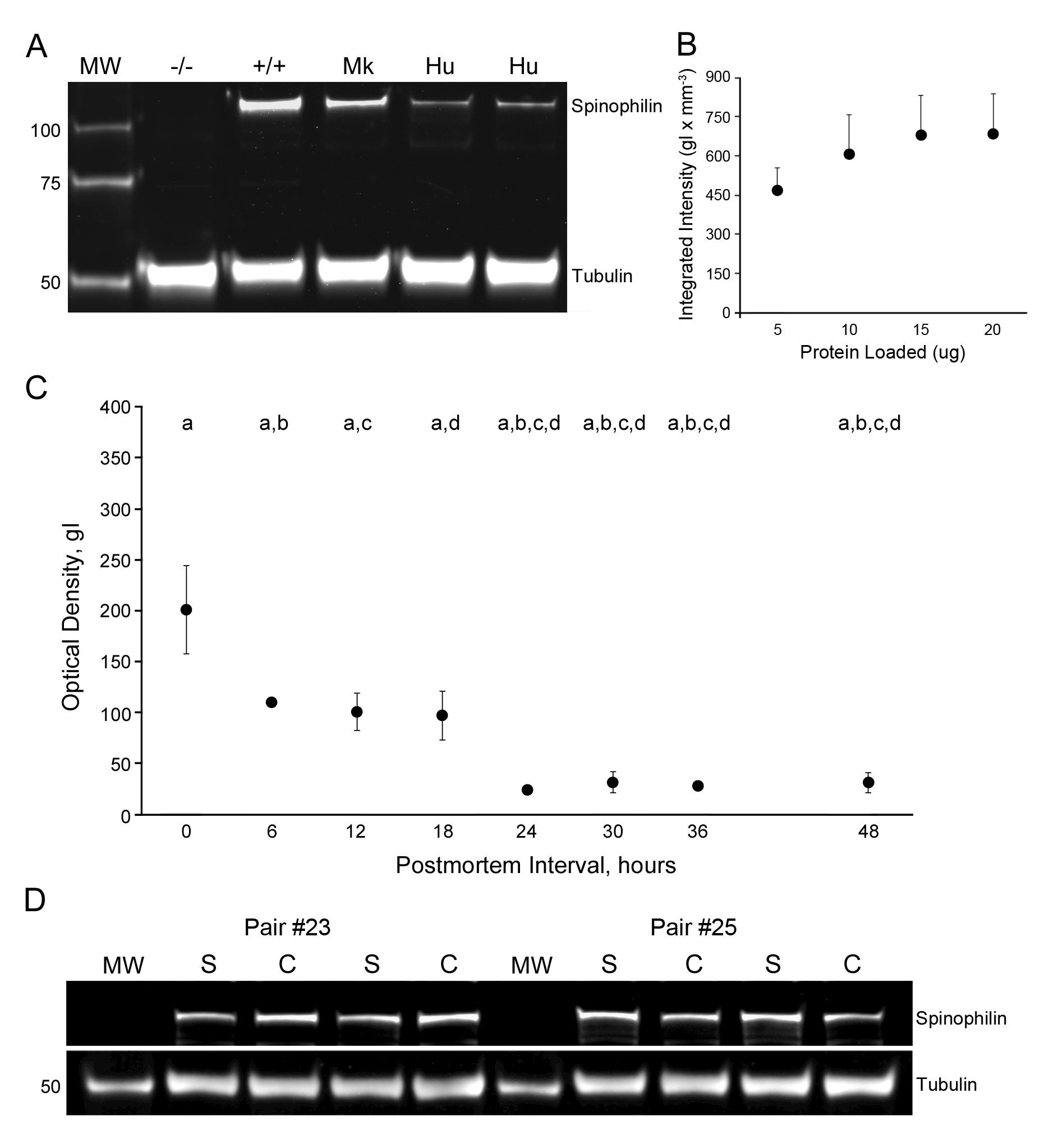

Supplement: Figure S1 — Spinophilin Western Blot Characterization and Validation. A) Spinophilin immunoreactivity in human, wild type mouse (+/+) and spinophilin knockout mouse (−/−) demonstrate specificity of spinophilin detection. B) Spinophilin optical density as a function of micrograms of protein loaded per lane. The mean (SD) of a eight repeated assays of a human subject is shown. It can be seen that the protein loading used in the comparison of schizophrenia and control subjects (10 µg) sits within the linear detection range. C) Optical densities for spinophilin in mice (N = 2) in which the interval from sacrifice to brain harvesting was experimentally varied between 0 and 48 hours. Mean (SEM) value at each time point is shown. Time points sharing the same superscript letter differ significantly. D) Detection of spinophilin in auditory cortex gray matter extracts from two subject pairs. S, subjects with schizophrenia, C, matched control subjects. (TIF) [file pone.0059458.s001.tif]

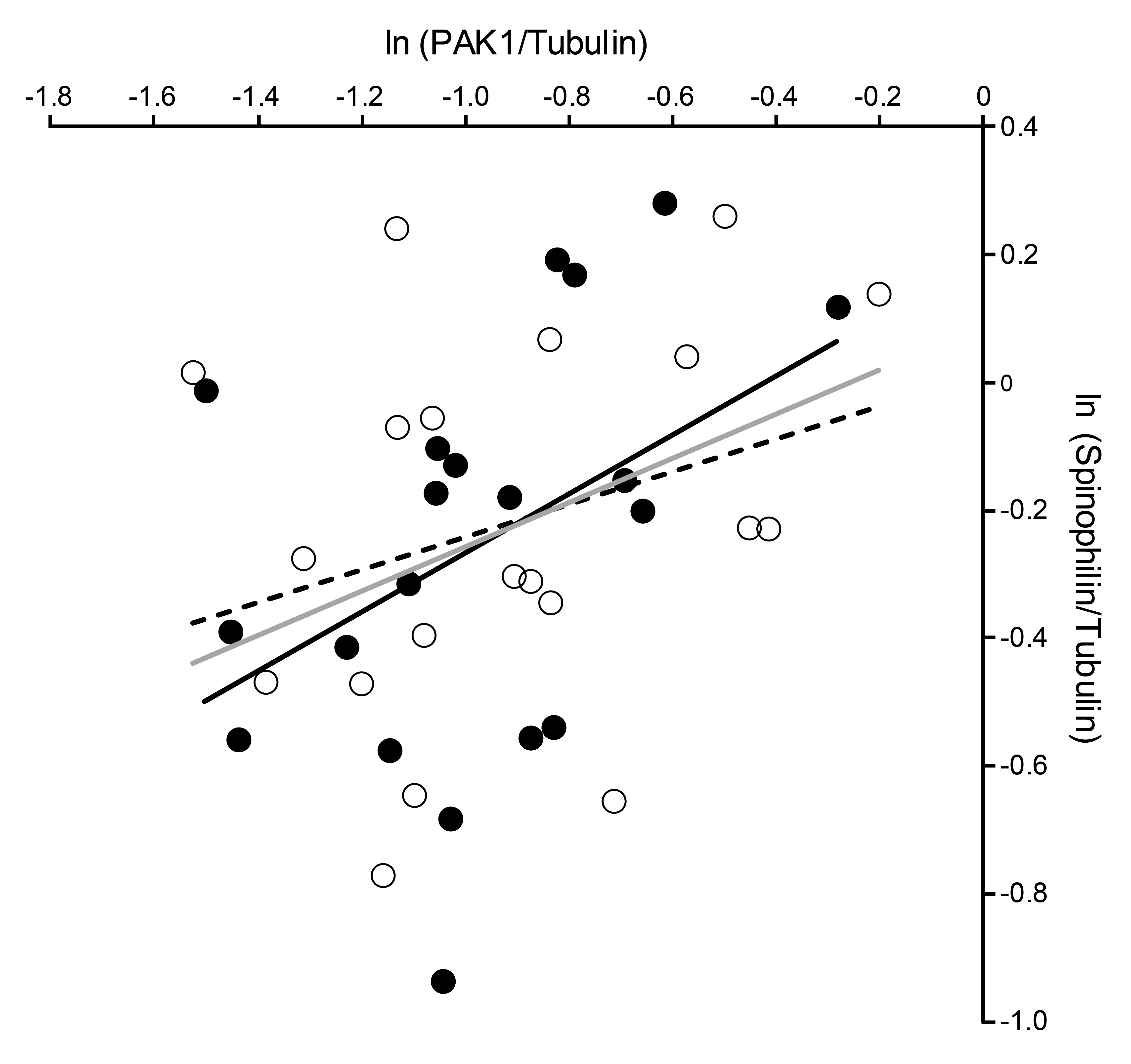

Supplement: Figure S2 — Correlation of PAK1 and Spinophilin Protein Levels within Subjects. Filled circles represent subjects with schizophrenia, open circles represent normal control subjects. Black line is the regression line for schizophrenia subjects (r = 0.43, p = 0.057). Dashed line is the regression line for control subjects (r = 0.30, p = 0.19). Gray line is the regression line for both groups combined (r = 0.37, p = 0.02). (TIF) [file pone.0059458.s002.tif]
